# Green Synthesised Carbon Nanodots Using the Maillard Reaction for the Rapid Detection of Elemental Selenium in Water and Carbonated Beverages

**DOI:** 10.3390/nano15151161

**Published:** 2025-07-28

**Authors:** Arjun Muthu, Duyen H. H. Nguyen, Aya Ferroudj, József Prokisch, Hassan El-Ramady, Chaima Neji, Áron Béni

**Affiliations:** 1 Doctoral School of Nutrition and Food Science, University of Debrecen, 4032 Debrecen, Hungary; arjun.muthu@agr.unideb.hu; 2 Institute of Agricultural Chemistry and Soil Science, Faculty of Agricultural and Food Sciences and Environmental Management, University of Debrecen, 138 Böszörményi Street, 4032 Debrecen, Hungary; beniaron@agr.unideb.hu; 3 Institute of Animal Science, Faculty of Agricultural and Food Sciences and Environmental Management, Biotechnology and Nature Conservation, University of Debrecen, 138 Böszörményi Street, 4032 Debrecen, Hungary; ferroudj.aya@agr.unideb.hu (A.F.); jprokisch@agr.unideb.hu (J.P.); hassan.elramady@agr.kfs.edu.eg (H.E.-R.); 4 Institute of Life Sciences, Vietnam Academy of Science and Technology, 9/621 Vo Nguyen Giap Street, Linh Trung Ward, Thu Duc City 721400, Ho Chi Minh, Vietnam; 5 Soil and Water Department, Faculty of Agriculture, Kafrelsheikh University, Kafr El-Sheikh 33516, Egypt; 6 Institute of Nutrition, Doctoral School of Nutrition and Food Science, Faculty of Agricultural and Food Sciences and Environmental Management, University of Debrecen, 138 Böszörményi Street, 4032 Debrecen, Hungary; neji.chaima@agr.unideb.hu

**Keywords:** nano-selenium, nano-sensors, fluorescence sensing, carbon dots

## Abstract

Selenium (Se) is an essential trace element involved in antioxidant redox regulation, thyroid hormone metabolism, and cancer prevention. Among its different forms, elemental selenium (Se^0^), particularly at the nanoscale, has gained growing attention in food, feed, and biomedical applications due to its lower toxicity and higher bioavailability compared to inorganic selenium species. However, the detection of Se^0^ in real samples remains challenging as current analytical methods are time-consuming, labour-intensive, and often unsuitable for rapid analysis. In this study, we developed a method for rapidly measuring Se^0^ using carbon nanodots (CNDs) produced from the Maillard reaction between glucose and glycine. The fabricated CNDs were water-dispersible and strongly fluorescent, with an average particle size of 3.90 ± 1.36 nm. Comprehensive characterisation by transmission electron microscopy (TEM), Fourier-transform infrared spectroscopy (FTIR), fluorescence spectroscopy, and Raman spectroscopy confirmed their structural and optical properties. The CNDs were employed as fluorescent probes for the selective detection of Se^0^. The sensor showed a wide linear detection range (0–12.665 mmol L^−1^), with a low detection limit (LOD) of 0.381 mmol L^−1^ and a quantification limit (LOQ) of 0.465 mmol L^−1^. Validation with spiked real samples—including ultra-pure water, tap water, and soft drinks—yielded high recoveries (98.6–108.1%) and low relative standard deviations (<3.4%). These results highlight the potential of CNDs as a simple, reliable, and environmentally friendly sensing platform for trace-level Se^0^ detection in complex food and beverage matrices.

## 1. Introduction

A crucial trace element found in plants, arthropods, and other organisms is selenium (Se). Selenium has many biological roles that benefit human health, such as controlling hormone release, combating heavy metals, scavenging free radicals, and reducing cancer [1,2,3]. Selenium compounds may lessen the incidence of cancer cell resistance according to research on ovarian cancer [4]. Elemental selenium (Se^0^) can alter the autophagy signalling mechanism, which can make it a potential cancer drug [5]. There are several species of selenium, including elemental selenium (Se^0^), organic selenium (selenomethionine, selenocysteine), and inorganic selenium (selenite, selenate). These are supplements for people deficient in selenium and adjuvant treatments for associated diseases [6,7]. Unlike many other micronutrients, selenium consumption varies significantly around the globe, with dangerous levels leading to paralysis, neurological and skin disorders, hair and nail loss, garlic breath, and poor dental health [8]. Elemental selenium or nano-selenium showed less sub-acute toxicity than other selenium species in mice [9]. Incorporating the nano-Se into the feed/food matrix is better since it has better bioavailability and less toxicity. Traditional techniques like Atomic Fluorescence Spectrometry (AFS) and Inductively Coupled-Plasma Mass Spectrometry (ICP-MS) are commonly employed for selenium speciation and quantification. It includes the detection of elemental selenium (Se^0^) in environmental and food samples [10,11]. However, these methods often require complex sample preparation steps such as acid digestion, chemical reduction, hydride generation, and filtration, followed by dilution with hydrochloric acid and gas–liquid separation. The entire process can take more than 4–5 h per sample [12,13,14,15]. Additionally, the equipment costs are high as AFS systems cost between $30,000 and $60,000, while ICP-MS devices can cost from $150,000 to $300,000, excluding the cost of consumables, trained personnel, and specialised infrastructure. Such requirements hinder their use for rapid, on-site testing and routine screening [16]. These issues highlight the urgent need for simpler, cost-effective, and faster detection methods for Se^0^ monitoring.

Fluorescent nanomaterials have garnered significant attention in recent years, surpassing other fluorescent dye probes. This increased interest is attributed to their wide-ranging applications in sensing various target analytes, printing inks, biological monitoring, and fields related to solar cells [17]. Carbon nanodots (CNDs) represent an innovative category of carbon nanomaterials characterised by their dimensions of less than 10 nm. They were initially found in 2004 using preparative electrophoresis to purify single-walled carbon nanotubes [18].

The primary factor contributing to the recent surge of interest in these minuscule CNDs is their remarkable fluorescence, leading to their classification as fluorescent carbon. Fluorescent nanomaterials can overcome several of the drawbacks of traditional fluorescent dyes, including low photostability, decreased fluorescence intensity, and rapid photobleaching, thanks to their unique properties, which include quantum size effects [19,20]. To detect contaminants such as pesticides [21,22], food additives [23,24], and heavy metals [25,26], CNDs have become an exciting component in the development of high-sensitivity probes. Additionally, they can be used in packaging materials to detect foodborne pathogens, identify food spoilage, and prolong the shelf life of food [27,28,29]. Interestingly, the CNDs can quickly detect and estimate other analytes, such as metal ions and organic molecules, for example, Mn (II) [30], Hg(II) [25,31], Fe(III) [32,33], Cd(II) [34], hydrogen peroxide [35], glucose [36], tetracycline [37], etc. Synthesising and developing new kinds of detection and estimation techniques using CNDs will be helpful in many analytical aspects [38]. It can improve the accuracy of diagnosis and increase the efficiency of detection and estimation of several analytes. Using CNDs has several significant benefits, some of which are listed below in specific essential terms.

(i) Green synthesis—CNDs can be synthesised via the simple hydrothermal reaction using Maillard precursors involving sugar and amino acids, which are both non-toxic, food-grade precursors. This method avoids hazardous reagents or solvents, making it environmentally sustainable and scalable [39,40].
(ii) Fluorescence stability—CNDs can exhibit excitation-dependent fluorescence with stable emission over a wide pH range (3–11), ionic strengths (0.1–1.0 mol L^−1^ NaCl), and incubation periods up to 60 min. These features ensure consistent performance across various environmental and sample conditions [41,42].
(iii) Biocompatibility—Using biologically safe materials and surface groups such as –OH, –COOH, and –NH_2_ improves water solubility and reduces toxicity, making CNDs suitable for use in food, beverages, and biological applications [43,44].

For the biocompatibility and safety of carbon nanodots (CNDs) we produced them using eco-friendly precursors, specifically glucose and glycine, through a Maillard reaction. This study had the following two main goals: (i) to characterise the synthesised CNDs using transmission electron microscopy (TEM), fluorescence spectroscopy, Raman spectroscopy, and Fourier-transform infrared (FTIR) spectroscopy, (ii) to assess the performance of these CNDs as fluorescent nano-sensors for detecting elemental selenium (Se^0^) in real-world matrices, including tap water and commercially available drinks. Additionally, the fluorescence-based sensing platform was rigorously evaluated for its stability, selectivity, and sensitivity to Se^0^, showing the potential of CNDs as reliable nanoprobes for monitoring trace-level selenium in complex environments.

## 2. Materials and Methods

### 2.1. Chemicals

Sodium Selenite, 37% hydrochloric acid, L-ascorbic acid, sodium hydroxide, and sodium chloride were procured from VWR, International Ltd. (Lutterworth, Leics, UK). Metal ion standards such as Ca^2+^, K^+^, Fe^3+^, Mg^2+^, Mn^2+^, Ni^2+^, Na^+^, and Zn^2+^ were purchased from Scharlab S.L. (Barcelona, Spain). Ultra-pure water was used throughout every experiment.

### 2.2. Characterisation

A transmission electron microscope (TEM) JEM-2000FXII (JEOL Ltd., Tokyo, Japan) was used to evaluate the shape and size of the particles of CNDs. For TEM sample preparation, a drop of diluted and 0.22 µm filtered CND solution was drop-cast onto a carbon-coated copper grid and dried at room temperature. A Lab RAM HR Evolution Confocal Raman Microscope (Horiba, Ltd., Kyoto, Japan) was used to measure the Raman spectra, and 532 nm excitation lasers were used for scanning. Solid CND freeze-dried powder was placed in the slide, and the Raman spectra were recorded. The fluorescence spectra were measured using a Spectrofluorometer FP-8500, which is situated in Jasco, OK, USA [45].

### 2.3. Synthesising of CNDs

CNDs were meticulously synthesised following the protocol of Ngugen et al., [45]. Glycine was mixed with glucose in an equimolar ratio (0.1 mmol L^−1^ of each precursor), and 20 mL of ultra-pure water was added. Based on previous reports, the equimolar ratio was chosen. This provides the optimal carbonisation and fluorescence yield with balanced sugar (carbon source) and amino acid (nitrogen source) precursors. The mixture was heated at 120 °C for 12 h and then cooled to room temperature. Equal aliquots of 40% cold ethanol were added and incubated for 30 min at −20 °C freezer. The mixture was centrifuged for 30 min at 5000× *g*, and the supernatant was collected. Later, using a 0.22 µm filter, the supernatant was filtered and dried using a freeze dryer. The obtained powder is used as carbon nanodots (CNDs). Se^0^ was prepared by Badger and Prokisch [46]. Selenite was mixed with ascorbic acid in a molar ratio of 1:10 in water. This green reduction reaction yields red Se^0^. A 0.22 µm filter was used to filter all samples before they were further examined.

### 2.4. CNDs Stability Analysis

To evaluate the stability of CNDs, fluorescence spectra were recorded at pH (3–11), duration (0–60 min), and NaCl content (0.1–1.0 mol L^−1^). An emission mode with a 5 nm emission bandwidth and a 2.5 nm exciting bandwidth was chosen. The measurement used an excitation wavelength of 360 nm, an emission range of 300–700 nm, and a scan speed of 1000 nm min^−1^. The temperature at which all samples were measured was 25 ± 0.1 °C.

### 2.5. Selectivity and Validation

To check the selectivity of the synthesised CNDs towards Se^0^, the interference studies were carried out with a range of potential interference ions. A 100 mg L^−1^ concentration was prepared for each metal ion solution, such as Ca^2+^, K^+^, Fe^3+^, Mg^2+^, Mn^2+^, Ni^2+^, Na^+^, and Zn^2+^. Each metal ion was mixed with and without CNDs individually under identical conditions, and the corresponding response was recorded. I_o_/I was calculated, where I_o_ corresponds to the intensity of the metal ion with CNDs and I corresponds to the intensity of the metal ion.

Se^0^ was detected using ultra-pure water, tap water from a laboratory faucet, and a soft drink (7up) from a supermarket (Aldi, Debrecen, Hungary) as real samples. The samples were tested using the spiking method, adding 126.6 µmol L^−1^ and 633.3 µmol L^−1^ of Se^0^, respectively. Three test runs were conducted, and the average of the results was determined.

### 2.6. Statistical Analysis

GraphPad Prism version 9.0 (San Diego, CA, USA) was used to analyse the statistical findings. The National Institutes of Health’s (Staten Island, NY, USA) ImageJ software version 1.54D was used to process the TEM and particle distribution images. All experimental results were expressed as mean ± standard deviation (n = 3). Tukey’s multiple comparison test was used to calculate the level of significance, where different alphabetical letters represent the level of significance, *p* < 0.001.

## 3. Results and Discussion

### 3.1. Characterisation of CNDs

**Figure 1:**
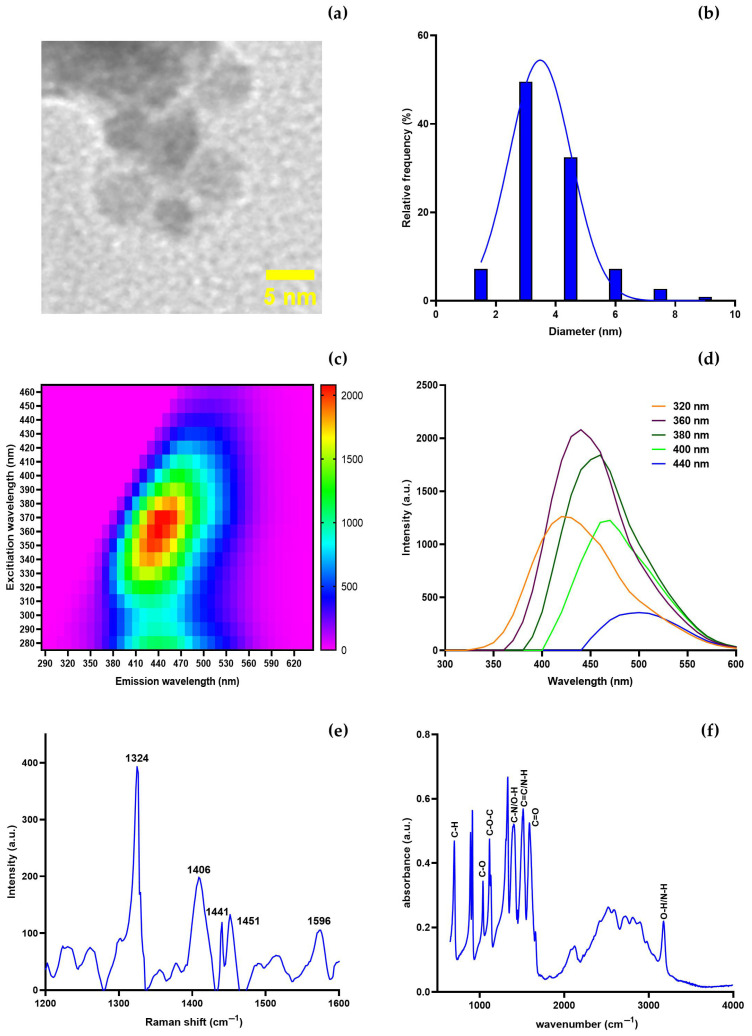
Characterisation of carbon nanodots (CNDs). (**a**) A transmission electron microscopy (TEM) image of CNDs at a scale of 5 nm. (**b**) A histogram with an average size of 3.90 ± 1.36 nm displays the particle size distribution. (**c**) CNDs’ three-dimensional fluorescence spectra. (**d**) CNDs two-dimensional emission–excitation–intensity fluorescence spectrum. (**e**) CNDs Raman spectra. (**f**) FTIR spectra of CNDs.

CNDs were prepared using the sample preparation protocol outlined in Section 2.3, and characterised by transmission electron microscopy (TEM), Fourier-transform infrared spectroscopy (FTIR), fluorescent spectroscopy, and Raman spectroscopy (Figure 1). TEM analysis showed that the CNDs are well-dispersed, spherical nanoparticles with a uniform morphology (Figure 1a). The particles lacked crystallinity, indicating their amorphous carbonaceous nature. The average particle diameter was calculated to be 3.90 ± 1.36 nm, which is consistent with previous reports [34,37,38]. Figure 1b shows the particle size distribution histogram, which confirms that most synthesised CNDs lie within the 2–5 nm range, with a sharp peak at around 3.5 nm. This narrow size distribution helps to ensure the CNDs have uniform optical properties and are readily dispersible in aqueous solutions. The photoluminescent behaviour of the CNDs was assessed using fluorescence spectroscopy. Three-dimensional spectra of CNDs (Figure 1c) show a characteristic excitation-dependent fluorescence pattern, with the highest intensity seen at an excitation wavelength of 360 nm and an emission maximum at around 430 nm. This attribute is due to multiple emissive traps or surface states introduced during the carbonisation process and surface functionalisation [47,48,49]. The two-dimensional spectra of the CNDs (Figure 1d) exhibit a clear red shift in the emission maximum as the excitation wavelength increases (from 320 to 440 nm), indicating a typical excitation-dependent emission profile. Such behaviour is often linked to heterogeneous energy states resulting from varying particle sizes, surface defects, or functional groups [50]. The excitation-dependent fluorescence makes these CNDs ideal candidates for sensing applications, where tuneable photoluminescence is beneficial [51].

Raman spectroscopy was conducted to investigate the carbon structure of the synthesised CNDs. As shown in Figure 1e, the following three significant peaks were observed: the D band at 1324 cm^−1^, the F band at 1406 cm^−1^, and the G band at 1596 cm^−1^. The D band corresponds to the breathing mode of sp^3^-hybridised carbon atoms and is indicative of structural defects or disorders in the graphitic framework. The bands at 1441 cm^−1^ and 1451 cm^−1^ are attributed to the vibrational modes of surface-bound functional groups, such as C–H bending (–CH_2_, –CH_3_) and C–N/C–O bonds, introduced during the Maillard reaction [52]. Usually, D bands situated at the 1330–1350 cm^−1^ range originate from defects and disorders within the carbon lattice and double resonant processes around the K point of the Brillouin Zone (BZ) boundary [53]. The G band arises from the E_2_g phonon of sp^2^-hybridised carbon atoms, reflecting the presence of graphitic domains [54,55]. The intermediate F band, centred around 1406 cm^−1^, is predominantly in the samples with high-intensity D bands. It has been attributed to additional structural disorder and surface functionalities such as amide or pyrrolic groups, likely introduced during the glycine-assisted synthesis [56,57]. The intensity ratio of the D to G bands (I_D_/I_G_) suggests that the CNDs possess a defect-rich, amorphous carbon structure conducive to surface reactivity and photoluminescence.

Fourier-transform infrared (FTIR) spectroscopy was used to identify the functional groups present on the CND surfaces (Figure 1f). A broad absorption band centred at ~3400 cm^−1^ corresponds to the O–H and N–H stretching vibrations, indicating the presence of hydroxyl and amine groups. Peaks at 2920 and 2850 cm^−1^ are attributed to asymmetric and symmetric C–H stretching vibrations from aliphatic chains. A distinct absorption peak at ~1720 cm^−1^ is indicative of C=O stretching from carboxylic acid or amide groups. The C=C stretching or N–H bending vibrations are observed near ~1600 cm^−1^, reflecting conjugated aromatic structures or peptide bonds. Peaks at 1400–1250 cm^−1^ correspond to C–N and C–O–C stretching, suggesting the presence of amines and ether linkages, while the bands below 1100 cm^−1^ are attributed to C–H bending modes of aromatic frameworks [58,59]. These findings confirm that the CNDs are functionalized with a variety of hydrophilic and chemically reactive moieties, such as hydroxyl, carboxyl, amine, and ether groups, which improve aqueous dispersibility and facilitate interactions with target analytes such as Se^0^ [60].

### 3.2. Stability of CNDs

**Figure 2:**
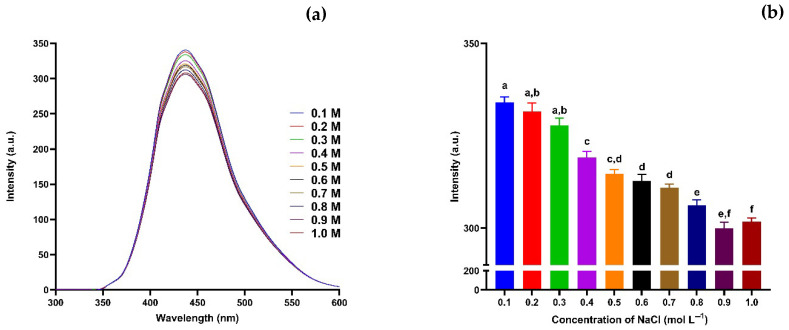
Stability of CNDs at NaCl 0.1–1.0 mol L^−1^. (**a**) CNDs with varying concentrations of NaCl at a 360 nm excitation wavelength and an emission wavelength range of 300–700 nm. (**b**) CNDs with different concentrations of NaCl at a 360 nm excitation wavelength and a 430 nm emission wavelength. Values are represented as mean ± standard deviation, where n = 3, and different alphabetical letters show the level of significance, *p* < 0.001.

Utilising the fluorescence of carbon nanodots (CNDs), making a nano-sensor detect Se^0^ through CNDs is possible. The suggested sensing system comprises CNDs at an appropriate concentration (0.100 mmol L^−1^) in an aqueous solution, where the CNDs function as fluorometric reporters. Additionally, the fluorescence stability of CNDs is evaluated in terms of pH, salt, and time. To assess the ionic strength stability of the synthesised carbon nanodots (CNDs), their fluorescence behaviour was evaluated in aqueous solutions containing various concentrations of NaCl, ranging from 0.1 to 1.0 mol L^−1^ (Figure 2a). Increasing the NaCl concentration resulted in a gradual decrease in fluorescence intensity at the maximum emission wavelength (~430 nm), suggesting that higher ionic strength leads to partial quenching of the CNDs emissions. Figure 2b shows a clearer picture of this trend, with statistically significant differences seen across the tested concentrations. At lower salt concentrations (0.1–0.3 mol L^−1^), the fluorescence intensity stayed relatively stable. However, above 0.4 mol L^−1^, a gradual decrease in emission intensity was observed, with fluorescence significantly reduced at concentrations of 0.6 mol L^−1^. At 1.0 mol L^−1^, the intensity dropped by over 30% compared to the control (0.1 mol L^−1^), suggesting salt-induced quenching and possible aggregation or shielding of emissive sites [61,62].

The decrease in fluorescence intensity results from electrostatic screening and surface charge effects. At low ionic strengths, strong electrostatic repulsion keeps CNDs dispersed, maintaining their optical properties [63]. As ionic strength rises, the electrical double layer around the CNDs compresses, lowering zeta potential and causing colloidal instability and partial aggregation [64]. This aggregation reduces the efficiency of radiative recombination because particles are closer together and may enable Förster resonance energy transfer (FRET) [65]. Moreover, Na^+^ ions shield negatively charged surface groups (–COOH, –OH), disrupting surface passivation and changing the emissive states. These factors lead to fluorescence quenching and demonstrate that CND’s behaviour depends on ionic strength [63]. These findings indicate that the CNDs retain their photostability under mild ionic conditions, but excessive ionic strength may mask surface charges or interfere with surface passivation, resulting in fluorescence quenching. The results show that the CNDs have moderate ionic tolerance, making them suitable for sensing applications in biologically and environmentally relevant matrices that typically have salt contents below 0.5 mol L^−1^ [66,67].

**Figure 3:**
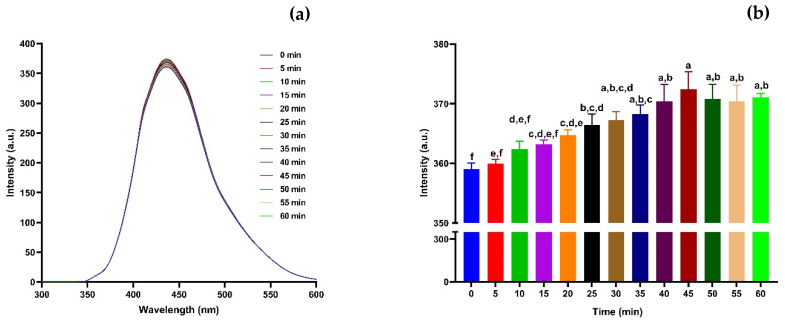
Stability of CNDs across different periods (0–60 min). (**a**) Stability of CNDs at different times (0–60 min) at 360 nm excitation wavelength and 300–700 nm emission wavelength. (**b**) Stability of CNDs at different times (0–60 min) at 360 nm excitation wavelength and 430 nm emission wavelength. All values are represented as mean ± standard deviation, where n = 3, and different alphabetical letters show level of significance, *p* < 0.001.

Further evaluation of the fluorescence stability of the CNDs was carried out over 60 min to assess their photostability in ambient aqueous conditions. The emission spectra recorded at different time intervals (Figure 3a) showed no significant shift in peak emission wavelength (~430 nm). Still, a gradual increase in fluorescence intensity was observed as the incubation time progressed. In Figure 3b, the fluorescence intensity increased steadily from 0 min to 45 min, with statistical analysis confirming notable differences among the various time points. At the initial time point (0 min), the lowest intensity was observed. The signal increased significantly within the first 30 min (*p* < 0.001), possibly due to improved surface hydration or the stabilisation of emissive traps on the CNDs surface [61]. The observed increase in emission intensity may be attributed to environmental stabilisation or the structural relaxation of surface functional groups, both of which enhance radiative recombination. After 45 min the CNDs maintained high fluorescence intensity with no significant decline, indicating robust temporal stability under constant excitation [68,69]. These findings support the high photostability and dispersion stability of the CNDs in aqueous media, a desirable feature for real-time sensing or long-term imaging applications. This result aligns with previous reports on CNDs exhibiting stable emission behaviour under continuous observation and prolonged exposure in aqueous matrices [70].

**Figure 4:**
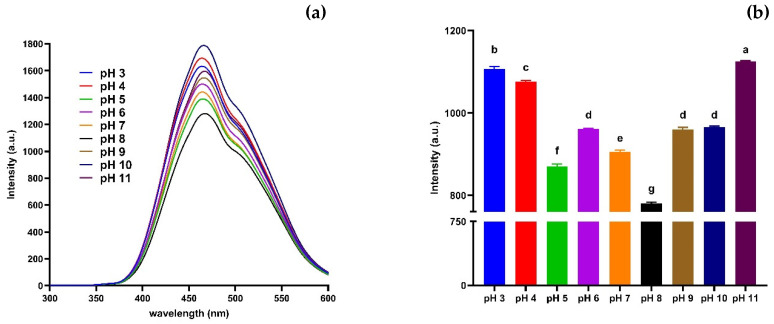
Stability of CND at different pH levels (3–11). (**a**) CNDs with different pH values (3–11) at 360 nm excitation wavelength and 300–700 nm emission wavelength. (**b**) CNDs with different pH values (3–11) at 360 nm excitation wavelength and 430 nm emission wavelength. Values are represented as mean ± standard deviation, where n = 3, and different alphabetical letters show level of significance *p* < 0.001.

To check their environmental adaptability and sensing potential, the fluorescence properties of CNDs over a wide range of pH values, from 3 to 11, were evaluated. As shown in Figure 4a, the CNDs emitted strong fluorescence across all pH values, with the emission peak remaining consistent at around 430 nm, indicating that the emissive centres are structurally stable. However, significant differences in fluorescence intensity were observed (Figure 4b). The intensity was highest at pH 11, followed by pH 3, suggesting that the electronic environment or surface state is optimised under both acidic and basic conditions.

At neutral pH (7) the lowest fluorescence was recorded, indicating a substantial quenching effect. The initial increase in fluorescence at pH 3–4 may be due to the protonation of surface amine or hydroxyl groups, which reduces non-radiative pathways. Conversely, the decline around pH 6–7 suggests increased competition from protonated/deprotonated species, which may disrupt surface passivation or enhance dynamic quenching [50]. The recovery of fluorescence at pH 10–11 could be due to deprotonation-induced electron delocalisation or aggregation-induced emission effects in alkaline conditions [71]. Overall, the results show that the CNDs exhibit pH-sensitive fluorescence with tuneable intensity but retain spectral stability, indicating suitability for biosensing or environmental applications where pH varies [72]. These findings are consistent with previous studies reporting that the photoluminescence of CNDs is modulated by the protonation–deprotonation equilibria of surface functional groups such as –COOH, –NH_2_, and –OH [28,29].

Based on the findings, the CNDs produced in this study demonstrated remarkable fluorescence stability, which is essential for ensuring accurate and reliable outcomes in Se^0^ detection in feed, food, and beverage industries [73].

### 3.3. Calibration and Validation of CNDs for the Measurement of Se^0^

**Figure 5:**
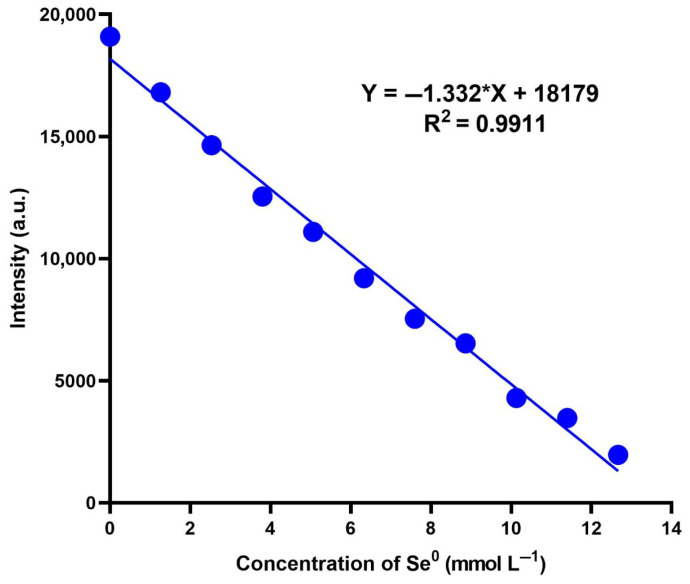
The standard linear calibration curve of Se^0^ from 0 to 12.665 mmol L^−1^, where *p* < 0.0001.

**Figure 6:**
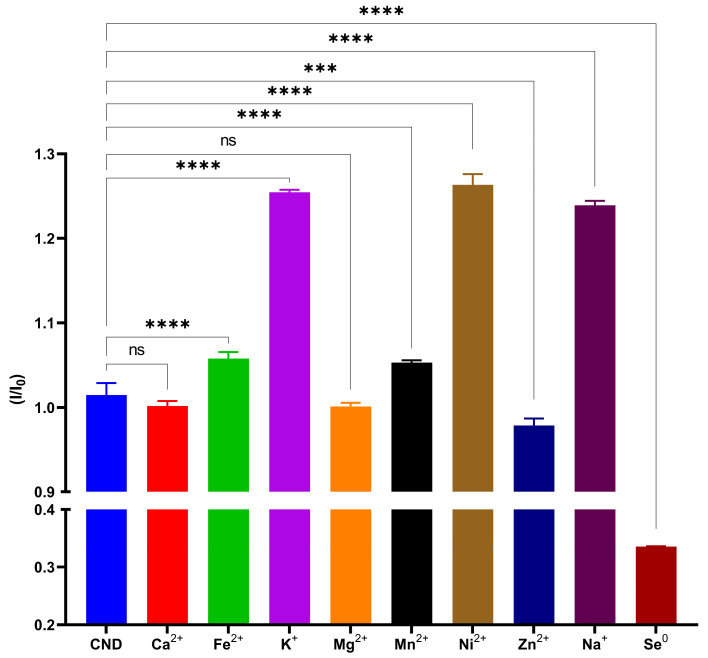
Selectivity of CNDs. Measures of 100 mg L^−1^ of Ca^2+^, Fe^2+^, K^+^, Mg^2+^, Mn^2+^, Ni^2+^, Zn^2+^, and Na^+^, compared Se^0^. Data are expressed as mean ± SD (n = 3). Statistical significance was determined using one-way ANOVA followed by Tukey’s post hoc test: *** = *p* < 0.001, **** = *p* < 0.0001 which indicates the level of significance; ns = not significant.

Standard curves were made by measuring the intensity of the fluorescence spectrum at a 360 nm excitation wavelength for various Se^0^ concentrations. On the day of the measurement, a new standard curve was created. Over a ten-point calibration range that includes concentrations ranging from 0 to 12.665 mmol L^−1^, the linear calibration line for Se^0^ was established (Figure 5). The measurement has a repeatability of less than 0.1%. The method’s determined detection limit was 0.381 mmol L^−1^. The European Medicines Agency and AOAC International validation processes assessed this approach’s precision, reproducibility, daily variation, and linearity [74,75]. The regression line, Y = −1.332*X + 18,179, created the standard curve, producing an R^2^ score of 0.9911. According to the findings the limit of detection (LOD) was 0.381 mmol L^−1^ and the limit of quantitation (LOQ) was 0.465 mmol L^−1^. The selectivity of the CNDs was evaluated by exposing them to a range of common metal ions, each at a concentration of 100 mg L^−1^ (Figure 6). The fluorescence response was expressed as the ratio I/I_o_ (where I denotes the fluorescence intensity of the CNDs with the analyte and I_0_ is the intensity in the absence of the analyte), which was recorded for Ca^2+^, Fe^2+^, K^+^, Mg^2+^, Mn^2+^, Ni^2+^, Zn^2+^, and Na^+^ and compared against Se^0^. Among all the tested species, Se^0^ had a significant decrease in fluorescence intensity, resulting in the lowest I/I_0_ ratio (~0.34), indicating strong quenching and the high selectivity of CNDs for Se^0^. This response suggests a particular interaction between Se^0^ and the surface functional groups of the CNDs, possibly involving charge-transfer or redox-based quenching mechanisms. In contrast, most metal ions exhibited negligible or even enhancing effects on fluorescence intensity [76]. Notably, K^+^, Ni^2+^, and Na^+^ produced a statistically significant increase in I/I_0_, implying that these ions may stabilise the excited state of CNDs through electrostatic interactions [77]. Metal ions such as Ca^2+^, Fe^2+^, Mg^2+^, and Zn^2+^ caused minor changes; these findings indicate that the CNDs fluorescence remains stable in the presence of physiologically or environmentally relevant concentrations of common metal ions, a desirable trait for selective sensing. These results suggest CNDs’ high specificity towards Se^0^ [78].

To assess the practical applicability of the developed CND-based fluorescent sensor, spiking and recovery experiments were conducted using ultra-pure water, tap water, and a commercial soft drink matrix. Se^0^ was added at two concentrations (126.6 µmol L^−1^ and 633.3 µmol L^−1^), and the corresponding recovery percentages and relative standard deviations (RSDs) were recorded. As shown in Table 1, the CNDs have demonstrated excellent recovery ranging from 98.60% to 108.1%, with low RSD values (0.2–3.4%) indicating high precision and reproducibility. Specifically, ultra-pure and tap water samples showed recoveries above 99%, while recoveries in soft drink matrices remained consistent with acceptable RSDs below 1.1%. The statistical groupings (a, b) suggest no significant deviation, which shows CNDs’ stability across different sample types. The sample was represented as the measure in the average of three replicates. A comparison with existing Se^0^ detection techniques is presented in Table 2. Conventional methods such as hydride-generation AFS provide excellent sensitivity (LOD-0.00152 μmol L^−1^ for biological samples) [79] and colorimetric assays using AuNPs reach an LOD of 0.05 μmol L^−1^ [80]. Electrochemical-based sensors offer sensitivity with an LOD-0.00152 μmol L^−1^ with limited ranges of 0.1267–0.6332 μmol L^−1^. Our Maillard-synthesised CNDs probe achieves a lower LOD (0.381 mmol L^−1^), a broader dynamic range, green synthesis, and robustness in beverage matrices. It is important to note that the current method targets elemental selenium (Se^0^), which is not regulated in the same way as inorganic selenium species. The WHO guideline value for total selenium in drinking water is 0.127 µmol L^−1^ (0.01 mg L^−1^), applying to selenate and selenite [81]. However, Se^0^ concentrations in fortified or processed samples, such as Se^0^-enriched beverages or feed formulations, can be significantly higher. The linear detection range of 0 to 12.665 mmol L^−1^ used in this study accounts for such scenarios, demonstrating the method’s reliability. With proper dilution or preconcentration, this platform can be adapted for detecting lower concentrations.

**Figure 7:**
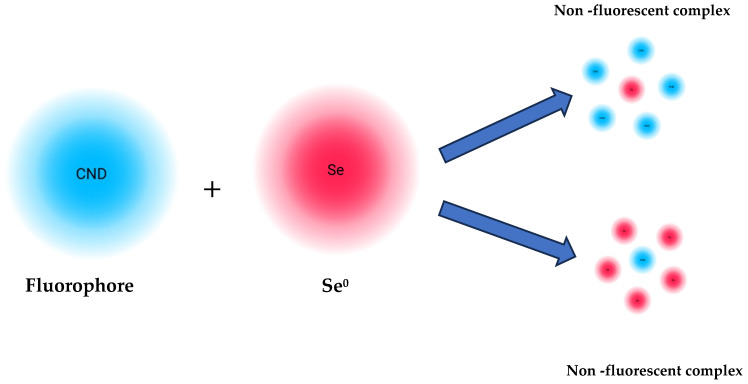
Mechanism of fluorescence quenching between CNDs and Se0.

In a hypothetical scenario of static quenching, if a non-fluorescent ground state complex developed between the fluorophore and quencher then the fluorescence lifetime (τ_0_/τ = 1) would not change (where τ_0_ and τ denote the lifetime without and with the quencher, respectively), which is shown in Figure 7 [49,50]. Accordingly, a dynamic quenching process is suggested by any change in the lifetime [82]. According to Mahajan et al. [83], the decrease in lifespan suggests that quenching is dynamic and that CNDs and Se^0^ spontaneously interact through a radiative emission pathway in excited states. An excited state interaction with the excited nanoparticles is predicted when Se^0^ molecules are close to surface-charged CNDs. This is explained by an electron or energy transfer process that causes quenching due to chelation. The creation of the complex at excited states, which transforms into fluorescence quenching by increasing Se^0^ concentrations, was thus suggested by the decrease in the fluorescence lifetime of CNDs in the presence of Se^0^. These findings are consistent with earlier reports on carbon dot-based fluorescence sensors, which have demonstrated robust performance in complex matrices with minimal interference [84]. The high recovery values and low variability further highlight the reliability of the proposed method for trace-level selenium determination in real-world samples. Such performance is crucial for environmental and food safety monitoring, where matrix effects often compromise analytical accuracy [85].

**Table 2:** Comparison of previously available methods.

Method	LOD (μmol L^−1^)	Linear Range (μmol L^−1^)	Green Synthesis and Biocompatibility	Sample Matrix	Ref.
Hydride generation (HG-AFS)	0.00152	>0.4052	No	Biological samples	[79]
Hydride generation with AuNPs and absorption with AgNPs	0.05	0.4–4.0	No	Biological and environmental sample	[80]
Electrochemical sensor with AuNPs	0.00152	0.1267–0.6334	No	Seawater samples	[86]
Fluorescence quenching with CNDs	381	0–12,665	yes	Water, tap water, and soft drink	This study

## 4. Conclusions

A green, simple, and scalable method was used to synthesise carbon nanodots (CNDs) from glucose and glycine using the Maillard reaction. The CNDs showed excellent structural, chemical, and optical properties, including strong excitation-dependent fluorescence, nanoscale size (3.90 ± 1.36 nm), and surface functional groups that promote analyte interaction. These features enable their use as effective fluorescent nanoprobes for detecting elemental selenium (Se^0^). The sensing platform proved stable across a wide pH range, various ionic strengths, and extended incubation periods, demonstrating the environmental adaptability of the CNDs. It shows a detection limit of 0.381 mmol L^−1^, a limit of quantification with 0.465 mmol L^−1^, and high sensitivity, with the CNDs displaying strong selectivity for Se^0^ over common metal ions. Validation with real samples using ultra-pure water, tap water, and soft drinks showed high recovery rates (98.60% to 108.1%) and reproducibility (0.2–3.4%), highlighting the practical feasibility of this method. Overall, these findings establish CNDs as a promising, cost-effective, and environmentally friendly alternative for detecting trace-level selenium. The method has significant potential for use in environmental monitoring, food safety testing, and biomedical diagnostics.

## Figures and Tables

**Table 1:** The concentration of Se^0^ in real samples.

Sample	Spiked Concentration of Se^0^ (µmol L^−1^)	Recovery (%)	RSD (%)
S1 Ultra-pure H_2_O	126.6	103.8 ± 3.5 ^a,b^	3.4
S2 Ultra-pure H_2_O	633.3	98.60 ± 0.7 ^b^	0.7
S3 Tap H_2_O	126.6	102.9 ± 0.2 ^a,b^	0.2
S4 Tap H_2_O	633.3	108.1 ± 0.4 ^a^	0.3
S5 soft drink	126.6	99.40 ± 0.1 ^b^	1.1
S6 soft drink	633.3	100.7 ± 0.6 ^b^	0.6

Values represented as mean ± standard deviation (n = 3). Where a represents *p* < 0.001. Superscripts a and b represent samples that show a statistically significant difference between the samples.

## Data Availability

Data will be made available by the authors on request.
